# Global Incidence of Neurological Manifestations Among Patients Hospitalized With COVID-19—A Report for the GCS-NeuroCOVID Consortium and the ENERGY Consortium

**DOI:** 10.1001/jamanetworkopen.2021.12131

**Published:** 2021-05-11

**Authors:** Sherry H.-Y. Chou, Ettore Beghi, Raimund Helbok, Elena Moro, Joshua Sampson, Valeria Altamirano, Shraddha Mainali, Claudio Bassetti, Jose I. Suarez, Molly McNett

**Affiliations:** 1 Department of Critical Care Medicine, University of Pittsburgh School of Medicine, Pittsburgh, Pennsylvania; 2 Department of Neurology and Department of Neurosurgery, University of Pittsburgh School of Medicine, Pittsburgh, Pennsylvania; 3 Laboratorio di Malattie Neurologiche, Istituto di Ricerche Farmacologiche Mario Negri IRCCS, Milano, Italy; 4 Department of Neurology, Neurocritical Care Unit, Medical University of Innsbruck, Innsbruck, Austria; 5 Division of Neurology, Centre Hospitalier Universitaire of Grenoble, Grenoble Alpes University, Grenoble Institute of Neuroscience, Grenoble, France; 6 Division of Cancer Epidemiology and Genetics, National Cancer Institute, Baltimore, Maryland; 7 Department of Neurology, The Ohio State University, Columbus; 8 Department of Neurology, University of Bern, Inselspital, Bern, Switzerland; 9 Departments of Anesthesiology and Critical Care Medicine, Neurology, and Neurosurgery, The Johns Hopkins University School of Medicine, Baltimore, Maryland; 10 College of Nursing, The Ohio State University, Columbus

## Abstract

**Importance:**

The COVID-19 pandemic continues to affect millions of people globally, with increasing reports of neurological manifestations but limited data on their incidence and associations with outcome.

**Objective:**

To determine the neurological phenotypes, incidence, and outcomes among adults hospitalized with COVID-19.

**Design, Setting, and Participants:**

This cohort study included patients with clinically diagnosed or laboratory-confirmed COVID-19 at 28 centers, representing 13 countries and 4 continents. The study was performed by the Global Consortium Study of Neurologic Dysfunction in COVID-19 (GCS-NeuroCOVID) from March 1 to September 30, 2020, and the European Academy of Neurology (EAN) Neuro-COVID Registry (ENERGY) from March to October 2020. Three cohorts were included: (1) the GCS-NeuroCOVID all COVID-19 cohort (n = 3055), which included consecutive hospitalized patients with COVID-19 with and without neurological manifestations; (2) the GCS-NeuroCOVID COVID-19 neurological cohort (n = 475), which comprised consecutive patients hospitalized with COVID-19 who had confirmed neurological manifestations; and (3) the ENERGY cohort (n = 214), which included patients with COVID-19 who received formal neurological consultation.

**Exposures:**

Clinically diagnosed or laboratory-confirmed COVID-19.

**Main Outcomes and Measures:**

Neurological phenotypes were classified as self-reported symptoms or neurological signs and/or syndromes assessed by clinical evaluation. Composite incidence was reported for groups with at least 1 neurological manifestation. The main outcome measure was in-hospital mortality.

**Results:**

Of the 3055 patients in the all COVID-19 cohort, 1742 (57%) were men, and the mean age was 59.9 years (95% CI, 59.3-60.6 years). Of the 475 patients in the COVID-19 neurological cohort, 262 (55%) were men, and the mean age was 62.6 years (95% CI, 61.1-64.1 years). Of the 214 patients in the ENERGY cohort, 133 (62%) were men, and the mean age was 67 years (95% CI, 52-78 years). A total of 3083 of 3743 patients (82%) across cohorts had any neurological manifestation (self-reported neurological symptoms and/or clinically captured neurological sign and/or syndrome). The most common self-reported symptoms included headache (1385 of 3732 patients [37%]) and anosmia or ageusia (977 of 3700 patients [26%]). The most prevalent neurological signs and/or syndromes were acute encephalopathy (1845 of 3740 patients [49%]), coma (649 of 3737 patients [17%]), and stroke (222 of 3737 patients [6%]), while meningitis and/or encephalitis were rare (19 of 3741 patients [0.5%]). Presence of clinically captured neurologic signs and/or syndromes was associated with increased risk of in-hospital death (adjusted odds ratio [aOR], 5.99; 95% CI, 4.33-8.28) after adjusting for study site, age, sex, race, and ethnicity. Presence of preexisting neurological disorders (aOR, 2.23; 95% CI, 1.80-2.75) was associated with increased risk of developing neurological signs and/or syndromes with COVID-19.

**Conclusions and Relevance:**

In this multicohort study, neurological manifestations were prevalent among patients hospitalized with COVID-19 and were associated with higher in-hospital mortality. Preexisting neurological disorders were associated with increased risk of developing neurological signs and/or syndromes in COVID-19.

## Key Points

**Question:**

What are the incidence of and outcomes associated with neurologic manifestations in patients with COVID-19?

**Findings:**

In this cohort study of 3744 patients in 2 large consortia, neurological manifestations were found in approximately 80% of patients hospitalized with COVID-19; the most common self-reported symptoms included headache (37%) and anosmia or ageusia (26%), whereas the most common neurological signs and/or syndromes were acute encephalopathy (49%), coma (17%), and stroke (6%). Presence of clinically captured neurologic signs and/or syndromes was associated with increased risk of in-hospital death.

**Meaning:**

These findings suggest that neurological manifestations are prevalent among patients hospitalized with COVID-19 and are associated with higher in-hospital mortality.

## Teaser

This cohort study investigates the neurological phenotypes, incidence, and outcomes among adults hospitalized with COVID-19.

## Introduction

The COVID-19 pandemic continues to affect millions of people globally, with increasing burdens of morbidity and mortality. More reports describe concomitant COVID-19 neurological manifestations,^1,2,3,4,5^ including symptoms such as headache, myalgia, anosmia, and ageusia as well as neurological syndromes such as encephalopathy, stroke, and coma, among others.^1,6,7,8,9,10,11,12,13,14,15,16,17^ Reports comprise case series^8,9,10,18^ and single and/or regional cohorts^4,19,20,21,22,23^ with varying data definitions, limiting the ability to accurately estimate the incidence of COVID-19 neurological manifestations and precluding data pooling and generalization across populations.^24,25,26^

To address this critical knowledge gap, 2 global consortia collaborated to establish worldwide incidence, type, and outcomes of neurological manifestations among patients hospitalized with COVID-19.^24,25,27,28^ The primary aims of this study were to (1) determine the incidence of COVID-19 neurological manifestations among global cohorts of hospitalized patients and (2) investigate the association of neurological manifestations with in-hospital mortality. A secondary exploratory aim was to identify potential risk factors associated with the development of neurological symptoms and signs or syndromes.

## Methods

### Study Design

#### Cohorts

The study population was derived from 2 large consortia: (1) the Global Consortium Study of Neurologic Dysfunction in COVID-19 (GCS-NeuroCOVID), a large multicenter cohort study set up to systematically establish the incidence, severity, and outcomes of neurological manifestations among patients with COVID-19,^27,28^ and (2) the European Academy of Neurology (EAN) Neuro-COVID Registry (ENERGY), a prospective registry established to evaluate the incidence of COVID-19 neurological manifestations and their outcomes at 6 and 12 months.^24^ The central coordinating center of the GCS-NeuroCOVID established ethics approval for a multicenter study from the University of Pittsburgh, confirmed local ethics approval with a waiver of informed consent because of the observational nature of the study, and executed data use agreements with each participating site. All centers that participated in the ENERGY Consortium received ethics committee approval, and all patients in the ENERGY cohort provided written or verbal informed consent, depending on requirements at each site. This study follows the Strengthening the Reporting of Observational Studies in Epidemiology (STROBE) reporting guideline.

The GCS-NeuroCOVID and ENERGY consortia have complementary global geographic reach and established a formal partnership to harmonize common data elements in their respective case report forms (eTable 1 in the Supplement 1) and combine data in a general analysis of the global and regional burden of COVID-19 neurological manifestations.^25^

Three separate cohorts were included in this study: (1) the GCS-NeuroCOVID all COVID-19 cohort (3055 patients at 6 sites), which included consecutive patients hospitalized with COVID-19 with and without neurological manifestations; (2) the GCS-NeuroCOVID COVID-19 neurological cohort (475 patients at 9 sites), which included consecutive patients hospitalized with COVID-19 and neurological manifestations; and (3) the ENERGY registry (214 patients at 13 sites), which included patients with COVID-19 and neurological manifestations.

#### GCS-NeuroCOVID

The design, rationale, and data elements of GCS-NeuroCOVID study have been previously published.^27,28^ The setting included secondary or tertiary centers caring for patients hospitalized with COVID-19. Inclusion criteria were patients aged 18 or older admitted to an acute care hospital with clinically diagnosed or laboratory-confirmed COVID-19. Exclusion criteria were patients with severe preexisting neurological dysfunction, such as baseline coma or vegetative state, that precluded determination of new neurological dysfunction. Race and ethnicity were recorded by local investigators based on patient self-identification during routine clinical care, history, and clinical examination. The GCS-NeuroCOVID Consortium included 2 separate cohorts. The all COVID-19 cohort included consecutive eligible patients over a 3-month study period regardless of neurological manifestations. The COVID-19 neurological cohort included consecutive eligible patients who were identified as having neurological manifestations over a 3-month study period. Both cohorts used the same standardized case report form.

As part of the pragmatic design to promote feasibility, data elements were divided into core and supplemental sections. Core elements were a robust minimal data set available regardless of care settings and resource availability, posed minimal data collection burden on frontline clinicians, and were mandatory from all sites. Supplemental data elements captured additional clinical features, and reporting was encouraged but optional. By design, all data elements were available through history and clinical observation and were not dependent on diagnostic testing availability, which may exhibit center-specific variabilities due to service disruptions during a pandemic.^28^

#### ENERGY

The procedures of the European registry are detailed in a study protocol administered to participating study sites.^29,30^ The setting included primary, secondary, and tertiary care centers providing care to patients with COVID-19. Study inclusion criteria were as follows: aged 18 years or older, clinically diagnosed COVID-19, case ascertainment through neurological consultation, and patient informed consent. Neurologist members of the EAN or its affiliated national societies registered consecutive patients fulfilling the inclusion criteria. Race/ethnicity were not recorded, as most registered patients were White individuals; however, patients from populations with other ethnic origins were confirmed by the local investigator. The ENERGY study included both hospitalized and nonhospitalized patients; however, in harmonization with the GCS-NeuroCOVID study, only hospitalized patient data were reported for this study. ENERGY data elements captured general symptoms and signs and prominent comorbidities of COVID-19 as well as neurological symptoms, signs, or diagnoses.

### Neurological Manifestations

Neurological manifestations were differentiated into (1) self-reported symptoms (ie, headache, anosmia and ageusia, history of syncope) and (2) neurological signs or diagnoses captured by clinical evaluation (ie, acute encephalopathy, stroke, coma, seizure and/or status epilepticus, dysautonomia, meningitis and/or encephalitis, myelopathy, plegia and/or paralysis, aphasia, movement abnormalities, abnormal tone, abnormal brainstem reflexes, and sensory abnormalities). Overall incidence of neurological manifestations was reported in 2 composite groups: the all neurological group included any patient with at least 1 self-reported neurological symptom and/or clinically captured neurological sign or syndrome. To minimize ascertainment bias resulting from self-reported symptoms, a separate signs and syndromes group was defined to include patients with at least 1 clinically captured neurological sign or syndrome.

### Statistical Analysis

Descriptive statistics were provided for demographic and clinical characteristics of each cohort, including incidence of composite and individual neurological manifestations. We reported the total sample size available for each core and supplemental data element. No imputation was performed for missing data. Due to the emergent nature of COVID-19 pandemic, there was no formal a priori sample size and power calculations. Because the ENERGY and GCS-NeuroCOVID COVID-19 neurological cohorts did not include the general hospitalized COVID-19 population, subsequent analyses of associations used only the GCS-NeuroCOVID all COVID-19 cohort to examine the association of neurological signs and/or syndromes with outcome at hospital discharge.

We first evaluated the associations between demographic characteristics and neurological manifestations with the risk of in-hospital death using logistic regression and reported odds ratios (ORs) with 95% CIs from unadjusted and adjusted models. Models adjusted for known baseline factors associated with COVID-19 mortality, ie, study center, age, age squared (higher order terms were not statistically significant), sex, race, and ethnicity. Presence of multicollinearity was assessed using the variance inflation factor. Because 1 of the 6 centers in the GCS-NeuroCOVID all COVID-19 cohort did not record body mass index (BMI; calculated as weight in kilograms divided by height in meters squared), we only adjusted for BMI in secondary analyses. Because only 8 patients (0.3%) were missing 1 or more of the adjustment covariates, we performed a complete case analysis.

We then explored the associations between demographic characteristics and preexisting neurological conditions with the risk of developing clinically captured neurological signs and/or syndromes using logistic regression. We report ORs and 95% CIs from unadjusted models and models that adjusted for baseline characteristics (ie, age, age squared, sex, race, and ethnicity). The null hypothesis was rejected with *P* < .05. All tests were 2-tailed. All analyses were performed using JMP Pro version 14.0 (SAS Institute).

## Results

### Population Characteristics

Characteristics of all 3 cohorts are summarized in Table 1. The GCS-NeuroCOVID all COVID-19 cohort included 3055 consecutive patients (1742 [57%] men; mean age, 59.9 years [95% CI, 59.3-60.6 years]) hospitalized with COVID-19 with and without neurological manifestations from 6 sites in the US. The GCS-NeuroCOVID COVID-19 neurological cohort included 475 consecutive patients (262 [55%] men; mean age, 62.6 years [95% CI, 61.1-64.1 years]) hospitalized with COVID-19 with neurological manifestations from 9 sites in the US and Egypt. The ENERGY registry included 214 patients (133 [62%] men; mean age, 67 years [95% CI, 52-78 years]) from 13 sites in Europe, Asia and the Middle East, and Africa.

**Table 1.** Study Population Characteristics, Stratified by Cohort^a^

Characteristic	Patients, No./total No. (%)		
	EAN ENERGY Registry (n = 214)	GCS-NeuroCOVID Cohort	
		COVID-19 neurological (n = 475)	All COVID-19 (n = 3055)
Sites, No.	13^b^	9	6
Geographic regions			
United States			
Northeast	0	34/475 (7)	2221/3055 (73)
Midwest	0	283/475 (60)	834/3055 (27)
South	0	99/475 (21)	0
Europe	167/214 (78)	0	0
Asia	30 (14)	0	0
Africa	17 (8)	59/475 (12)	0
Demographic characteristics			
Sex			
Men	133/214 (62)	262/475 (55)	1742/3055 (57)
Women	81/214 (38)	213/475 (45)	1313/3055 (43)
Age, mean (95% CI), y	67 (52-78)	62.6 (61.1-64.1)	59.9 (59.3-60.6)
Race			
White	NA	207/475 (44)	1474/3054 (48)
Black or African American	NA	134/475 (28)	696/3054 (23)
Asian	NA	16/475 (3)	133/3054 (4)
Other^c^	NA	98/475 (21)	572/3054 (19)
Unknown	NA	20/475 (4)	179/3054 (6)
Hispanic ethnicity			
Yes	1/45 (2)	52/473 (11)	697/3053 (23)
No	44/45 (98)	421/473 (89)	2356/3053 (77)
BMI, median (IQR)	26 (23-29)	29.5 (24.6-35.7)	29.7 (25.8-34.5)
Risk factors and past medical history			
Preexisting neurological disorders	84/186 (45)	172/475 (36)	713/3055 (23)
Diabetes^d^	51/154 (33)	208/411 (51)	284/822 (35)
Coronary artery disease^d^	68/213 (32)	101/411 (25)	146/822 (18)
Hypertension^d^	107/160 (67)	273/411 (66)	473/822 (58)
Cerebrovascular disease^d^	39/214 (18)	55/411 (13)	87/821 (11)
Immunosuppressed^d^	13/213 (6)	41/411 (10)	139/822 (17)
Lung disease^d^	31/214 (14)	102/410 (25)	169/822 (21)
Smoking^d^	20/213 (9)	61/411 (15)	183/818 (22)
Treatment intensity			
ECMO	3/214 (1)	2/475 (<1)	40/3053 (1)
Dialysis	23/183 (13)	70/425 (16)	92/3054 (3)
Mechanical ventilation	44/214 (21)	186/475 (39)	867/3052 (28)
Hospital outcomes			
Hospital LOS, median (IQR), d^e^	23 (9-39)	10 (5-18)	8 (4-15)
Death	30/209 (14)	113/460 (25)	417/3049 (14)

Abbreviations: BMI, body mass index (calculated as weight in kilograms divided by height in meters squared); EAN, European Academy of Neurology; ECMO, extracorporeal membrane oxygenation; ENERGY, European Academy of Neurology Neuro-COVID Registry; GCS-NeuroCOVID, Global Consortium Study of Neurological Manifestations of COVID-19; IQR, interquartile range; LOS, length of stay; NA, not available.

^a^ The EAN ENERGY registry included patients with COVID-19 and neurological features. The COVID-19 neurological cohort included patients with COVID-19 and neurological features. The all COVID-19 cohort included patients with COVID-19, with and without neurological features.

^b^ Countries represented in the EAN ENERGY registry included Austria, Hungary, Portugal, Italy, Turkey, Switzerland, Tunisia, France, Egypt, Israel, and Ukraine.

^c^ Other races included American Indian, Pacific Islander, unknown, and not recorded.

^d^ Supplemental data elements capture additional clinical features and baseline risk factors. Reporting of supplemental data elements is optional, with some but not all sites reporting these data.

^e^ Data on hospital LOS were available for 29 patients (14%) in the ENERGY cohort, 392 (83%) in the COVID-19 neurological cohort, and 780 patients (26%) in the all COVID-19 cohort.

The cohorts consisted of predominantly White individuals (all COVID-19: 1474 [48%]; COVID-19 neurological: 207 [44%]); men; patients with overweight, ie, BMI greater than 25 (median [interquartile range] BMI, all COVID-19: 29.7 [25.8-34.5]; COVID-19 neurological: 29.5 [24.6-35.7]; ENERGY: 26 [23-29]); and older patients. All 3 cohorts had a high incidence of underlying medical conditions, such as diabetes, hypertension, and preexisting neurologic disorders. The proportion of patients receiving mechanical ventilation ranged from 44 of 214 patients (21%) in the ENERGY cohort to 186 of 475 patients (39%) in the COVID-19 neurological cohort. In-hospital mortality ranged from 30 of 209 patients (14%) in the ENERGY cohort to 113 of 460 patients (25%) in the COVID-19 neurological cohort.

### Incidence of Neurological Manifestations

We separately report the incidence of neurological manifestations for each cohort, as their different entry requirements may affect observed calculated estimates. Only the GCS-NeuroCOVID all COVID-19 cohort approximately reflected the general hospitalized COVID-19 population based on entry criteria. All core data elements for neurological symptoms or clinically captured signs and/or syndromes are reported with less than 5% missing data from the GCS-NeuroCOVID study cohorts and less than 10% missing data from ENERGY registry (Table 2). Only a subset of study sites reported supplemental data elements, which are available for 45 patients (21%) in the ENERGY cohort, 411 patients (87%) in the COVID-19 neurological cohort, and 822 patients (27%) in the all COVID-19 cohort.

**Table 2.** Neurologic Manifestations in Study Population, Stratified by Cohort^a^

Manifestation	Patients, No./total No. (%)		
	EAN ENERGY Registry (n = 214)	GCS-NeuroCOVID Cohort	
		COVID-19 neurological (n = 475)	All COVID-19 (n = 3055)
Neurological manifestations			
Any neurological manifestation^b^	169/214 (79)	475/475 (100)	2439/3054 (80)
Clinically captured signs or syndromes^c^	151/169 (89)	385/475 (81)	1628/3055 (53)
Self-reported neurological symptoms			
Headache	56/204 (27)	164/475 (35)	1165/3053 (38)
Anosmia or ageusia	46/199 (23)	91/449 (20)	840/3052 (28)
Syncope	4/212 (2)	58/475 (12)	152/3054 (5)
Clinically verified neurological signs or syndromes			
Acute encephalopathy	50/212 (24)	254/475 (53)	1541/3053 (50)
Stroke, all types	40/208 (19)	88/475 (19)	94/3054 (3)
Coma	22/214 (10)	118/475 (25)	509/3048 (17)
Seizure and/or status epilepticus	17/213 (8)	46/475 (10)	33/3053 (1)
Dysautonomia	16/197 (8)	20/475 (4)	37/3053 (1)
Meningitis and/or encephalitis	5/213 (2)	10/475 (2)	4/3053 (<1)
Myelopathy	6/213 (3)	13/474 (3)	6/3049 (<2)
Plegia and/or paralysis^d^	2/45 (4)	48/411 (12)	25/819 (3)
Aphasia^d^	0	47/411 (11)	15/308 (5)
New movement abnormalities^d^	NA	19/411 (5)	24/814 (3)
Abnormal tone^d^	2/45 (4)	51/411 (12)	14/304 (4)
Abnormal brainstem reflexes^d^	1/44 (2)	21/352 (6)	23/282 (8)
Sensory abnormalities^c^	1/45 (2)	51/411 (12)	15/812 (2)

Abbreviations: EAN, European Academy of Neurology; ENERGY, European Academy of Neurology Neuro-COVID Registry; GCS-NeuroCOVID, Global Consortium Study of Neurological Manifestations of COVID-19; NA, not available.

^a^ The EAN ENERGY registry included patients with COVID-19 and neurological features. The COVID-19 neurological cohort included patients with COVID-19 and neurological features. The all COVID-19 cohort included patients with COVID-19, with and without neurological features.

^b^ Presence of any self-reported neurological symptoms or clinically verified neurological signs or syndromes.

^c^ Presence of any clinically verified neurological sign or syndrome.

^d^ Supplemental data elements capture additional clinical features and baseline risk factors. Reporting of supplemental data elements is optional with some but not all sites reporting these data.

Across all 3 cohorts, headache was the most common self-reported symptom (Table 2), with 1165 of 3053 patients (38%) in the all COVID-19 cohort, 164 of 475 patients (35%) in the COVID-19 neurological cohort, and 56 of 204 patients (27%) in the ENERGY cohort, resulting in pooled incidence of 37% (1385 of 3732 patients). Self-reported anosmia or ageusia was also common, with 840 of 3052 patients (28%) in the all COVID-19 cohort, 91 of 449 (20%) in the COVID-19 neurological cohort, and 46 of 199 (23%) in the ENERGY cohort. Across cohorts, overall incidence of this symptom was 26% (977 of 3700 patients). History of syncope was the least common symptom, with an incidence of 152 of 3054 (5%) in the all COVID-19 cohort.

Acute encephalopathy was the most common clinically captured neurological sign and/or syndrome in all cohorts (1845 of 3740 patients [49%]), with 1541 of 3053 patients (50%) in the all COVID-19 cohort, 254 of 475 patients (53%) in the COVID-19 neurological cohort, and 50 of 212 patients (24%) in the ENERGY cohort. The next most common neurological signs and/or syndromes were coma and stroke. The incidence of coma was 509 of 3049 patients (17%) in the all COVID-19 cohort, while coma was reported in 22 of 214 patients (10%) in the ENERGY cohort and 118 of 475 (25%) in the COVID-19 neurological cohort, resulting in a pooled estimate of 17% (649 of 3737 patients) across all groups. A total of 94 of 3054 patients (3%) had a stroke. In contrast, the 2 neurologically focused cohorts both reported a higher incidence of stroke, with 88 of 475 patients (19%) in the COVID-19 neurological cohort and 40 of 208 patients (19%) in the ENERGY cohort. The least common neurological signs or syndromes were meningitis and/or encephalitis (4 of 3053 patients [0.1%] in all COVID-19 cohort; pooled estimate, 0.5% [19 of 3741]) and myelopathy (6 of 3049 patients [0.2%] in all COVID-19 cohort), an observation consistent across all 3 cohorts (eFigure in Supplement 1).

Across all cohorts, 3083 of 3743 patients (82%) across cohorts had any neurological manifestation. In the all COVID-19 cohort, 2439 of 3054 patients (80%) had any neurological manifestation, either self-reported and/or clinically captured (ie, the all neurological group). Excluding self-reported symptoms, incidence of any clinically captured neurological sign or syndrome (ie, the signs or syndromes group) was 53% (1628 of 3055 patients) in the all COVID-19 cohort and between 81% and 89% in the other 2 neurologically focused cohorts (385 of 475 patients [81%] in COVID-19 neurological; 151 of 169 [89%] in ENERGY).

In the all COVID-19 cohort, 1098 of 1312 women (84%) had any neurological manifestation, and 1341 of 1742 men (77%) had any neurological manifestation (Table 3). A total of 969 of 1742 men (56%) and 659 of 1313 women (50%) had clinically captured neurological signs or syndromes; incidence steadily increased across age groups, from 158 of 452 patients (35%) younger than 40 years to 307 of 398 patients (77%) older than 80 years. Self-reported symptoms such as headache and anosmia were more common in women than in men (headache: 581 of 1312 [44%] vs 584 of 1741 [34%]; anosmia or ageusia: 411 of 1311 [31%] vs 429 of 1741 [25%]), and their incidence decreased with age, from 223 of 452 (49%) in those younger than 40 years to 90 of 397 (23%) in those older than 80 years. Anosmia or ageusia similarly decreased from 184 of 452 (41%) in those younger than 40 years to 266 of 1136 (23%) in those older than 80 years. Acute encephalopathy was present in 916 of 1741 men (53%) and 625 of 1312 women (48%), and its incidence increased steadily with age, from 151 of 452 (33%) in those younger than 40 years to 293 of 397 (74%) in those older than 80 years.

**Table 3.** Incidence of Neurological Manifestations by Sex and Age in the All COVID-19 Cohort

Manifestation	No. of events/No. of individuals (%)					
	Sex		Age group, y			
	Male (n = 1742)	Female (n = 1313)	<40 (n = 452)	40-60 (n = 1059)	61-80 (n = 1136)	>80 (n = 398)
Overall groupings						
Any neurological manifestation^a^	1341/1742 (77)	1098/1312 (84)	368/452 (81)	813/1059 (77)	909/1136 (80)	343/397 (86)
Clinically captured signs or syndromes^b^	969/1742 (56)	659/1313 (50)	158/452 (35)	473/1059 (45)	689/1136 (61)	307/398 (77)
Self-reported neurological symptoms						
Headache	584/1741 (34)	581/1312 (44)	223/452 (49)	477/1058 (45)	371/1161 (33)	90/397 (23)
Anosmia or ageusia	429/1741 (25)	411/1311 (31)	184/452 (41)	299/1057 (28)	266/1136 (23)	90/397(23)
Syncope	96/1742 (6)	56/1312 (4)	11/452 (2)	47/1059 (4)	64/1136 (6)	30/397 (8)
Clinically verified neurological signs or syndromes						
Acute encephalopathy	916/1741 (53)	625/1312 (48)	151/452 (33)	439/1058 (41)	657/1136 (58)	293/397 (74)
Stroke, all types	53/1742 (3)	41/1312 (3)	4/452 (1)	31/1059 (3)	44/1136 (4)	15/397 (4)
Coma	331/1736 (19)	178/1312 (14)	51/452 (11)	172/1056 (16)	234/1133 (21)	52/397 (13)
Seizure and/or status epilepticus	19/1742 (1)	14/1311 (1)	4/452 (1)	12/1059 (1)	15/1136 (1)	2/396 (1)
Dysautonomia	24/1742 (1)	13/1311 (1)	7/452 (2)	10/1059 (1)	17/1135 (1)	3/397 (1)
Meningitis and/or encephalitis	3/1741 (<1)	1/1312 (<1)	0	2/1058 (<1)	1/1136 (<1)	1/397 (<1)
Myelopathy	6/1738 (<1)	0	1/452 (<1)	4/1056 (<1)	1/1134 (<1)	0
Plegia and/or paralysis^c^	16/462 (3)	9/357 (3)	2/104 (2)	10/294 (3)	11/317 (3)	2/101 (2)
Aphasia^c^	9/180 (5)	6/128 (5)	2/24 (8)	4/109 (4)	6/127 (5)	3/45 (7)
New movement abnormalities^c^	14/459 (3)	12/355 (3)	2/102 (2)	11/292 (4)	10/317 (3)	3/100 (3)
Abnormal tone^c^	5/179 (3)	10/125 (8)	1/23 (4)	5/108 (5)	8/125 (6)	1/45 (2)
Abnormal brainstem reflexes^c^	16/163 (10)	7/119 (6)	0	9/101 (9)	12/116 (10)	2/41 (5)
Sensory abnormalities^c^	12/461 (3)	3/351(1)	0	7/293 (2)	6/313 (2)	2/101 (2)

^a^ Presence of any self-reported neurological symptoms or clinically verified neurological signs or syndromes.

^b^ Presence of any clinically verified neurological sign or syndrome.

^c^ Supplemental data elements capture additional clinical features and baseline risk factors. Reporting of supplemental data elements is optional with some but not all sites reporting these data.

### Neurological Manifestations and In-Hospital Mortality

The presence of any neurological symptom or clinically captured sign or syndrome was associated with a higher risk of in-hospital death (OR, 1.77; 95% CI, 1.32-2.39) (Table 4; eTable 1 in Supplement 1). When limited to clinically captured neurologic signs or syndromes, the magnitude of the association with death was higher (OR, 6.41; 95% CI, 4.82-8.50). After adjusting for baseline differences by study site, age, sex, race, and ethnicity, the presence of any clinically captured neurologic sign or syndrome remained associated with an increased risk of death (aOR, 5.99; 95% CI, 4.33-8.28).

**Table 4.** Characteristics Associated With In-Hospital Death in All COVID-19 Cohort

Covariate	No. of deaths/No. of individuals (%)		Unadjusted		Adjusted^a^	
	Present	Absent	OR (95%CI)	*P* value	aOR (95% CI)	*P* value
Baseline characteristics						
Age, per 10-y increment	NA	NA	1.71 (1.59-1.84)	<.001	1.74 (1.60-1.88)	<.001
Male sex	270/1741 (16)	147/1308 (11)	1.45 (1.17-1.80)	<.001	1.66 (1.31-2.10)	<.001
Race^b^						
White	246/1469 (17)	0	1 [Reference]	NA	1 [Reference]	NA
Asian	10/132 (8)	0	0.41 (0.21-0.79)	.008	0.39 (0.20-0.77)	.007
African American	78/696 (11)	0	0.63 (0.48-0.82)	<.001	0.81 (0.61-1.09)	.16
Other^c^	58/572 (10)	0	0.56 (0.41-0.76)	<.001	1.05 (0.67-1.64)	.83
Unknown	25/179 (14)	0	0.81 (0.52-1.26)	.34	1.02 (0.63-1.64)	.94
Hispanic ethnicity	63/697 (9)	354/2351 (15)	0.56 (0.42-0.74)	<.001	0.86 (0.56-1.31)	.47
Preexisting neurological disorders	134/712 (19)	283/2337 (12)	1.68 (1.34-2.11)	<.001	1.19 (0.93-1.52)	.17
Overall neurological groupings, No. of events/No. of individuals (%)						
Any neurological manifestation^d^	362/2435 (15)	55/614 (9)	1.77 (1.32-2.39)	<.001	2.48 (1.70-3.62)	<.001
Clinically captured signs or syndromes^e^	357/1624 (22)	60/1425 (4)	6.41 (4.82-8.50)	<.001	5.99 (4.33-8.28)	<.001
Self-reported neurological symptoms, No. of events/No. of individuals (%)						
Headache	65/1161 (6)	352/1887 (19)	0.26 (0.20-0.34)	<.001	0.33 (0.24-0.44)	<.001
Anosmia or aguesia	89/840 (11)	328/2207 (15)	0.68 (0.53-0.87)	.002	0.81 (0.62-1.07)	.14
Syncope	7/151 (5)	410/2898 (14)	0.29 (0.14-0.63)	<.001	0.22 (0.10-0.48)	<.001
Neurologic signs or syndromes, No. of events/No. of individuals (%)						
Acute encephalopathy	340/1540 (22)	77/1508 (5)	5.27 (4.06-6.82)	<.001	5.51 (4.01-7.57)	<.001
Stroke, all types	22/94 (23)	395/2955 (13)	1.98 (1.21-3.23)	.001	1.60 (0.95-2.68)	.08
Coma	154/509 (30)	262/2534 (10)	3.76 (2.99-4.73)	<.001	7.70 (5.65-10.50)	<.001
Seizure and/or status epilepticus	6/33 (18)	411/3016 (14)	1.41 (0.58-3.43)	.45	1.54 (0.59-3.99)	.37
Meningitis and/or encephalitis	2/4 (50)	415/3044 (14)	6.33 (0.89-45.09)	.07	7.33 (0.80-67.42)	.08
Myelopathy	1/6 (17)	415/3038 (14)	1.26 (0.15-10.85)	.83	1.62 (0.17-15.09)	.67
Plegia and/or paralysis^f^	7/25 (28)	100/690 (13)	2.68 (1.09-6.59)	.046	1.50 (0.53-4.26)	.44
Aphasia^f^	6/15 (40)	57/289 (20)	2.71 (0.93-7.93)	.07	2.93 (0.91-9.41)	.07
Sensory abnormalities^f^	5/15 (33)	100/793 (13)	3.47 (1.16-10.3)	.04	1.12 (0.33-3.84)	.85
Abnormal brainstem reflexes^f^	18/23 (78)	39/255 (15)	19.94 (6.99-56.85)	<.001	24.28 (7.06-83.5)	<.001
Movement abnormalities^f^	4/26 (15)	101/784 (13)	1.23 (0.42-3.64)	.71	1.09 (0.34-3.47)	.89
Abnormal tone^f^	6/15 (40)	56/285 (20)	2.73 (0.93-7.98)	.07	4.53 (1.40-14.60)	.02

Abbreviations: aOR, adjusted odds ratio; NA, not applicable; OR, odds ratio.

^a^ Adjusted models include covariates study center, age, age squared (except when estimating the OR for age), sex, race, and ethnicity.

^b^ The *P *value for the overall group association of race with in-hospital was *P* < .001 for unadjusted models and *P* = .03 for adjusted models.

^c^ Other races included American Indian, Pacific Islander, unknown, and not recorded.

^d^ Presence of any self-reported neurological symptoms or clinically verified neurological signs or syndromes.

^e^ Presence of any clinically verified neurological sign or syndrome.

^f^ Supplemental data elements capture additional clinical features and baseline risk factors. Reporting of supplemental data elements is optional with some but not all sites reporting these data.

Self-reported neurological symptoms (ie, headache, anosmia or ageusia, syncope) were associated with a reduced risk of in-hospital death, while clinically captured neurologic signs or syndromes were associated with an increased risk of death (Table 4). After adjusting for baseline differences in study site, age, sex, race, and ethnicity, self-reported headache and syncope remained associated with a reduced risk of in-hospital death (headache: aOR, 0.33; 95% CI, 0.24-0.44; syncope: aOR, 0.22; 95% CI, 0.10-0.48). In contrast, clinically diagnosed neurological signs and/or syndromes, such as acute encephalopathy, coma, and abnormal brainstem reflexes, were associated with higher risk for in-hospital death (acute encephalopathy: aOR, 5.51; 95% CI, 4.01-7.57; coma: aOR, 7.70; 95% CI, 5.65-10.5; abnormal brainstem reflexes: aOR, 24.4; 95% CI, 7.06-83.5).

### Risk Factors for New Neurological Signs and Syndromes

Using data from the all COVID-19 cohort, we found that multiple baseline characteristics, including age, sex, race, ethnicity, and preexisting neurological disorders, were associated with an increased risk of developing clinically captured neurological sign or syndrome with COVID-19 (Table 5). In a model that mutually adjusted for these characteristics, older age (aOR per 10-year increment, 1.41; 95% CI, 1.34-1.48), male sex (aOR, 1.53; 1.30-1.82), White race (eg, Asian race vs White race: aOR, 0.62; 95% CI, 0.41-0.94), and preexisting neurological disorders (aOR, 2.23; 95% CI, 1.80-2.75) were associated with the risk of developing neurological signs or syndromes with COVID-19.

**Table 5.** Baseline Characteristics Associated With Presence of Clinically Verified Neurological Signs or Syndromes in the All COVID-19 Cohort

Covariate	Patients, No./total No. (%)		Unadjusted		Adjusted^a^	
	Present	Absent	OR (95%CI)	*P* value	aOR (95%CI)	*P* value
Age, per 10-y increment	NA	NA	1.39 (1.33-1.45)	<.001	1.41 (1.34-1.48)	<.001
Male sex	1004/1742 (58)	682/1313 (52)	1.26 (1.09-1.45)	.002	1.53 (1.30-1.82)	<.001
Race^b^						
White	886/1474 (60)	0	1 [Reference]	NA	1 [Reference]	NA
African American	324/696 (47)	0	0.41 (0.21-0.79)	<.001	0.80 (0.65-0.99)	.04
Asian	69/133 (52)	0	0.72 (0.50-1.02)	.03	0.62 (0.41-0.94)	.02
Other^c^	299/572 (52)	0	0.71 (0.59-0.87)	<.001	0.68 (0.51-0.90)	.008
Unknown	108/179 (60)	0	1.01 (0.74-1.39)	.80	0.99 (0.69-1.41)	.94
Hispanic ethnicity	340/697 (49)	1286/2356 (55)	0.79 (0.67-0.94)	.007	0.81 (0.62-1.05)	.11
Preexisting neurological disorder	511/713 (72)	1175/2342 (50)	2.51 (2.09-3.01)	<.001	2.23 (1.80-2.75)	<.001

Abbreviations: aOR, adjusted odds ratio; NA, not applicable; OR, odds ratio.

^a^ Adjusted models include covariates study center, age, age squared (except when estimating the OR for age), sex, race, and ethnicity.

^b^ The *P *value for the overall group association of race with in-hospital was *P* < .001 for unadjusted models and *P* = .008 for adjusted models.

^c^ Other races included American Indian, Pacific Islander, unknown, and not recorded.

## Discussion

To our knowledge, this is the first and largest study of neurological manifestations in patients hospitalized with COVID-19 across different cohorts and geographic regions. Neurological manifestations ranged from self-reported symptoms to more severe neurological signs or syndromes captured by clinical evaluation. Overall, 80% of these patients exhibited at least 1 new neurological symptom, sign, or syndrome, and 55% had at least 1 neurological sign or syndrome captured on clinical evaluation. We observed that acute encephalopathy was the most common neurological sign or syndrome with an incidence of 50% across the 3 separate cohorts, while meningitis and/or encephalitis and myelopathy were the least common, with incidences of 0.1% and 0.2%, respectively. Furthermore, the presence of neurological signs or syndromes with COVID-19 significantly increased the risk of dying during acute hospitalization after adjusting for variations by study site and baseline characteristics. Taken together, these observations highlight the importance of neurological manifestations in COVID-19 and their potential impact on disease outcome.

In addition to reporting global incidence of COVID-19 neurological manifestations, we reported the incidence of specific common neurological symptoms, signs, and syndromes across 3 different cohort types. This reporting was important, as it highlighted variations in rates based on region and cohort composition. Aggregating all data and presenting only pooled data could have resulted in inaccurate estimates and an overstatement of actual signs and syndromes. Thus, the presentation of the data as distinct cohorts allowed for precise reporting of parameters, which was needed to definitively build the science around these conditions within the setting of COVID-19 infection and to compare outcomes between patients with COVID-19 with and without neurological manifestations. Existing data from prior cohorts report incidence of acute encephalopathy ranging from 8% to 84%.^6,10,20,21,31,32,33^ In our large multicenter all COVID-19 patient cohort, we observed a 50% incidence of acute encephalopathy. Meanwhile, our 2 cohorts with neurological manifestation as entry criteria observed incidences of encephalopathy between 24% and 53%, likely reflecting differences in case ascertainment and severity of illness in the underlying populations. Acute encephalopathy carried a 5-fold higher risk of in-hospital death after adjusting for known risk factors, including age. This was consistent with another multicenter study^31^ showing acute encephalopathy in COVID-19 was more prevalent in older patients with medical comorbidities, with higher need for critical care and worse 30-day mortality.

The incidence of other common neurological syndromes, such as stroke and coma, varied among cohorts in our study. Existing studies with variable designs, sampling methods, and case definitions have reported wide ranges for stroke incidence, from 1% to 46%.^4,21,34,35^ Our all COVID-19 cohort observed stroke incidence at 3% in the hospitalized COVID-19 population, while our neurologically focused COVID-19 neurological cohort and ENERGY cohort reported a higher stroke incidence (19%), suggesting that clinical neurological syndromes, such as stroke, may be overrepresented in populations captured by neurological entry criteria. Coma among patients with COVID-19 has been reported,^36^ but its incidence is not known. Our study found coma incidence ranges from 10% to 25%. The higher incidence of acute encephalopathy and coma in GCS-NeuroCOVID cohorts compared with the ENERGY registry likely reflected differences in population characteristics, such as the proportion of patients receiving mechanical ventilation.

Measured incidence of self-reported neurological symptoms from our collective cohorts was within the range from existing literature: between 13% and 60% for headache^37^ and between 25% and 68% for anosmia or ageusia^38,39^ The observation that self-reported neurological symptoms, such as headaches, were associated with lower risk of death requires consideration. A separate retrospective study^38^ also found that headache was associated with lower mortality in 576 patients hospitalized with COVID-19. The observed association between self-reported neurological symptoms and lower mortality may reflect ascertainment bias, where such data may be insufficiently captured in patients with higher disease severity, particularly in the hospitalized COVID-19 population.

Consistent with existing reports, advanced age, male sex, White race, and higher BMI were associated with higher mortality.^36,40,41^ In addition, we found that presence of neurological signs or syndromes with COVID-19 carried a 5-fold higher risk for in-hospital death after adjusting for differences between study sites and baseline characteristics. It is important to note that we did not record the time or duration of the neurological observation and its association to in-hospital mortality. Particularly among those with advanced illness, this is an important consideration and should be included in future research. Despite this limitation, our findings were consistent with the growing evidence from smaller cohort studies^20,22,42,43,44,45^ and supported the conclusion that neurological manifestations with COVID-19 are an important risk factor for mortality. As such, a formal neurological consultation may be warranted when neurological signs or symptoms are suspected among individuals who test positive for COVID-19.

Our exploratory analysis found that in addition to baseline characteristics and underlying medical conditions, having a preexisting neurological disorder was independently associated with increased risk of developing neurological signs or syndromes with COVID-19. In our pragmatic study design, we did not categorize preexisting neurological disorders, nor did we classify potential etiologies of COVID-19 neurological signs or syndromes, which may have included exacerbation of existing neurological pathology, development of de novo neurological syndromes, or neurological complication of systemic illness.^36,46^ Additionally, less common neurological manifestations were recorded as supplemental data elements. Although the denominators in these initial estimates were small, as not all sites recorded this information, our preliminary findings highlighted possible presence of these phenotypes among hospitalized cohorts. To our knowledge, this was the first report to detail these syndromes, and it warrants additional investigation in future research on neurological manifestations in COVID-19. As such, these important questions will be addressed in future tiers of the GCS-NeuroCOVID consortium studies. Meanwhile, this observation provides a first general direction for identifying patients with COVID-19 who may be at higher risk of developing neurological signs or syndromes.

### Limitations

This study has limitations, including factors inherent in global cohort study design and data collection in the midst of a pandemic and the fact that this was not a formal population-based study. Both the GCS-NeuroCOVID and ENERGY consortia used a pragmatic design to facilitate rapid study launch and data collection by frontline clinicians, as traditional research resources were limited during pandemic surges. Both prospective and retrospective data capture were permitted, and this may have led to ascertainment bias. There were no a priori sample size and power calculations, and study sample size was based on available data from registered sites. Use of a global, multicenter approach may have resulted in heterogeneity of reporting due to clinical settings and interobserver variabilities. To limit infection exposure risk related to data collection, interrater reliability was not performed; however, all sites used standardized data definitions and assessment guides to minimize variability. Because of the variations in care delivery and research resource availability across global sites, our data set captures different cohort types. Some sites were able to provide a control cohort and denominator for calculating incidence of neurological manifestations by submitting data on all patients with COVID-19 patients, including those without neurological manifestations (ie, the GCS-NeuroCOVID all COVID-19 cohort). Other sites reported data solely among patients with COVID-19 and neurological manifestations (ie, GCS-NeuroCOVID COVID-19 neurological and ENERGY cohorts). While these differences limit the possibility of merging data across cohorts, inclusion of these different cohorts allowed for comprehensive reporting across different populations. Furthermore, our study only reports outcomes at hospital discharge and did not report detailed causes of death. Follow-up studies are underway to determine the associations of neurological manifestations with longer-term outcomes and to capture delayed onset neurological manifestations beyond acute hospitalization.

## Conclusions

This multicenter cohort study found that neurological manifestations in COVID-19 were highly prevalent and associated with premature mortality. Using a global network with standardized protocols and common data elements is critical to facilitate further studies to understand COVID-19 neurological manifestations, including disease progression, associations with long-term outcomes, pathobiological mechanisms, and societal impact.

## Notes

Supplement 1. **eTable 1.** Common Data Elements Across GCS NeuroCOVID and ENERGY Registry CRF **eTable 2.** Characteristics Associated With In-Hospital Death, Adjusted for Study Center, Age, Sex, Race, Ethnicity, and Body Mass Index for GCS-NeuroCOVID All COVID-19 Cohort **eFigure.** Prevalence of Clinically Captured Neurological Syndromes or Signs in Study Cohorts

Supplement 2. Nonauthor Collaborators
