# Supplementary material for: Global Incidence of Neurological Manifestations Among Patients Hospitalized With COVID-19—A Report for the GCS-NeuroCOVID Consortium and the ENERGY Consortium
Source: JAMA Netw Open. 2021 May 11;4(5):e2112131. doi: 10.1001/jamanetworkopen.2021.12131 (PMC8114143; doi:10.1001/jamanetworkopen.2021.12131)
Supplement: Supplement 1. — eTable 1. Common Data Elements Across GCS NeuroCOVID and ENERGY Registry CRF eTable 2. Characteristics Associated With In-Hospital Death, Adjusted for Study Center, Age, Sex, Race, Ethnicity, and Body Mass Index for GCS-NeuroCOVID All COVID-19 Cohort eFigure. Prevalence of Clinically Captured Neurological Syndromes or Signs in Study Cohorts [file jamanetwopen-e2112131-s001.pdf]

Supplemental Online Content

Chou SHY, Beghi E, Helbok R, et al; GCS-NeuroCOVID Consortium and ENERGY Consortium. Global incidence of neurological manifestations among patients hospitalized with COVID-19—a report for the GCS-NeuroCOVID Consortium and the ENERGY Consortium. *JAMA Netw Open*. 2021;4(5):e2112131. doi:10.1001/jamanetworkopen.2021.12131

eTable 1. Common Data Elements Across GCS NeuroCOVID and ENERGY Registry CRF
eTable 2. Characteristics Associated With In-Hospital Death, Adjusted for Study Center, Age, Sex, Race, Ethnicity, and Body Mass Index for GCS-NeuroCOVID All COVID-19 Cohort
eFigure. Prevalence of Clinically Captured Neurological Syndromes or Signs in Study Cohorts

This supplemental material has been provided by the authors to give readers additional information about their work.

© 2021 Chou SHY et al. *JAMA Network Open.*

eTable 1. Common Data Elements Across GCS NeuroCOVID and ENERGY Registry CRF

Variable	ENERGY Registry	GCS NeuroCOVID Cohorts
Center ID	x	x
Patient's code	x	x
Site of visit	x	x
Reason for neuro assessment	x	
Date of registration	x	x
Medical record #	x	x
Year of birth	x	x
Sex	x	x
Height	x	x
Weight	x	x
Smoking	x	x
Source of contact	x	
Date of symptom onset	x	x
Final COVID status	x	x
**Comorbidities**		
Arterial hypertension	x	x
Diabetes	x	x
Cardiovascular disease	x	x
Chronic kidney disease	x	
Chronic liver disease	x	
Chronic pulmonary disease	x	x
Anemia	x	
Cancer	x	x
Immunosuppressed state	x	x
Other non-neuro comorbidities	x	
Neuro comorbidities	x	x
Premorbid mRS	x	
**Complications**		
Dyspnea	x	
Pneumonia	x	x
Cardiovascular disease	x	x
Renal insufficiency/dialysis	x	x
Coagulation disease/DIC	x	
Refractory shock	x	x
ECMO	x	x
Hospital admission	x	x
ICU admission	x	x
Mechanical ventilation	x	x
**Neurological Findings**		
Headache	x	x

© 2021 Chou SHY et al. *JAMA Network Open.*

Hyposmia/hypogeusia	x	x
Dysautonomia	x	x
Vertigo	x	
Myalgia	x	
Myelopathy	x	x
Sleep disturbances	x	
Sleepiness/hypersomnia	x	
Cognitive impairment	x	
Dysexecutive syndrome	x	
Hyperactive delirium	x	x
Hypoactive delirium/acute encephalopathy	x	x
Stupor/coma	x	x
Syncope	x	x
Seizures/status epilepticus	x	x
Meningitis/encephalitis	x	x
Stroke	x	x
Sensory Abnormalities	x	x
Movement disorder/abnormalities	x	x
Ataxia	x	
Spinal cord disorder	x	x
Peripheral neuropathy	x	
Plegia/Paralysis		x
Abnormal brain stem reflexes	x	x
Aphasia	x	x
Abnormal tone	x	x
**Investigations and Outcomes**		
CSF	x	x
CT/MRI	x	x
Status at discharge	x	x
mRS at discharge	x	x
Date of death	x	x

© 2021 Chou SHY et al. *JAMA Network Open.*

eTable 2. Characteristics Associated With In-Hospital Death, Adjusted for Study Center, Age, Sex, Race, Ethnicity, and Body Mass Index for GCS-NeuroCOVID All COVID-19 Cohort

	Unadjusted		[a]Adjusted	
**Covariate**	**OR [95%CI]**	**p-value**	**aOR [95%CI]**	**p-value**
**Baseline Characteristics**				
**Age (per 10 yr)**	1.71 [1.59 -1.84]	<0.001	1.80 [1.65 – 1.96]	<0.001
**Male Sex**	1.45 [1.17 – 1.80]	<0.001	1.70 [1.33 – 2.18]	<0.001
**Body Mass Index (BMI)**	1.0 [0.98 – 1.01]	NS	1.01 [1.00 – 1.03]	0.030
**Race**	..	<0.001	..	0.040
**White**	1	..	1	..
**Asian**	0.41 [0.21 – 0.79]	0.0076	0.39 [0.19 – 0.80]	0.01
**African American**	0.63 [0.48 – 0.82]	<0.001	0.79 [0.57 – 1.10]	NS
**Other**	0.56 [0.41 – 0.76]	<0.001	1.13 [0.71 – 1.81]	NS
**Unknown**	0.81 [0.52 – 1.26]	NS	1.00 [0.61 – 1.65]	NS
**Hispanic Ethnicity**	0.56 [0.42 – 0.74]	<0.001	0.85 [0.54 – 1.33]	NS
**Pre-existing neurological disorders**	1.68 [1.34 – 2.11]	<0.001	1.22 [0.94 – 1.58]	NS
**Neurologic Manifestations**				
[b]**All Neuro +**	1.77 [1.32 – 2.39]	<0.001	1.79 [1.17 – 2.75]	0.006
[c]**Signs/Syndromes +**	6.41 [4.82 – 8.50]	<0.001	6.19 [4.24 – 9.02]	<0.001
**Self-Reported Neurological Symptoms**				
**Headache**	0.26 [0.20 – 0.34]	<0.001	0.31 [0.23 – 0.42]	<0.001
**Anosmia/Aguesia**	0.68 [0.53 – 0.87]	0.002	0.84 [0.63 – 1.11]	NS
**Syncope**	0.29 [0.14 – 0.63]	<0.001	0.13 [0.05 – 0.36]	<0.001
**Neurologic Signs/Syndromes**				
**Acute Encephalopathy**	5.27 [4.06 – 6.82]	<0.001	5.63 [3.92 – 8.08]	<0.001
**Stroke (all types)**	1.98 [1.21 – 3.23]	0.001	0.86 [0.44 – 1.69]	NS
**Coma**	3.76 [2.99 – 4.73]	<0.001	6.35 [4.59 – 8.77]	<0.001
**Seizure/Status Epilepticus**	1.41 [0.58 – 3.43]	NS	0.82 [0.23 – 2.95]	NS
**Meningitis/ Encephalitis**	6.33 [0.89 – 45.1]	NS	7.75 [0.82 – 73.0]	NS
**Myelopathy**	1.26 [0.15 – 10.8]	NS	0.85 [0.54 – 1.32]	NS
[d]**Plegia/Paralysis**	2.68 [1.09 – 6.59]	0.046	3.07 [0.81 – 11.7]	NS
[d]**Aphasia**	2.71 [0.93 – 7.93]	NS	3.84 [0.67 – 21.9]	NS
[d]**Sensory Abnormalities**	3.47 [1.16 – 10.3]	0.040	1.77 [0.15 – 21.5]	NS
[d]**Abnormal Brainstem Reflexes**	19.9 [6.99 – 56.9]	<0.001	23.6 [2.94 – 190]	0.002
[d]**Movement Abnormalities**	1.23 [0.42 – 3.64]	NS	1.21 [0.37 – 3.98]	NS
[d]**Abnormal Tone**	2.73 [0.93 – 7.98]	NS	3.89 [1.06 – 14.3]	0.049

Abbreviation: OR: Odds Ratio; aOR: Adjusted Odds Ratio; NS: p>0.05

[a]Adjusted models include covariates study center, age, age^2 (except when estimating the OR for age), sex, race, and ethnicity.

[b]All Neuro +: Presence of any self-reported neurological symptoms or clinically verified neurological signs or syndromes.

[c]Signs/Syndromes +: Clinically verified neurological signs or syndromes present.

[d]Supplemental data elements capture additional clinical features and baseline risk factors. Reporting of supplemental data elements is optional with some but not all sites reporting these data.

© 2021 Chou SHY et al. *JAMA Network Open.*

eFigure. Prevalence of Clinically Captured Neurological Syndromes or Signs in Study Cohorts

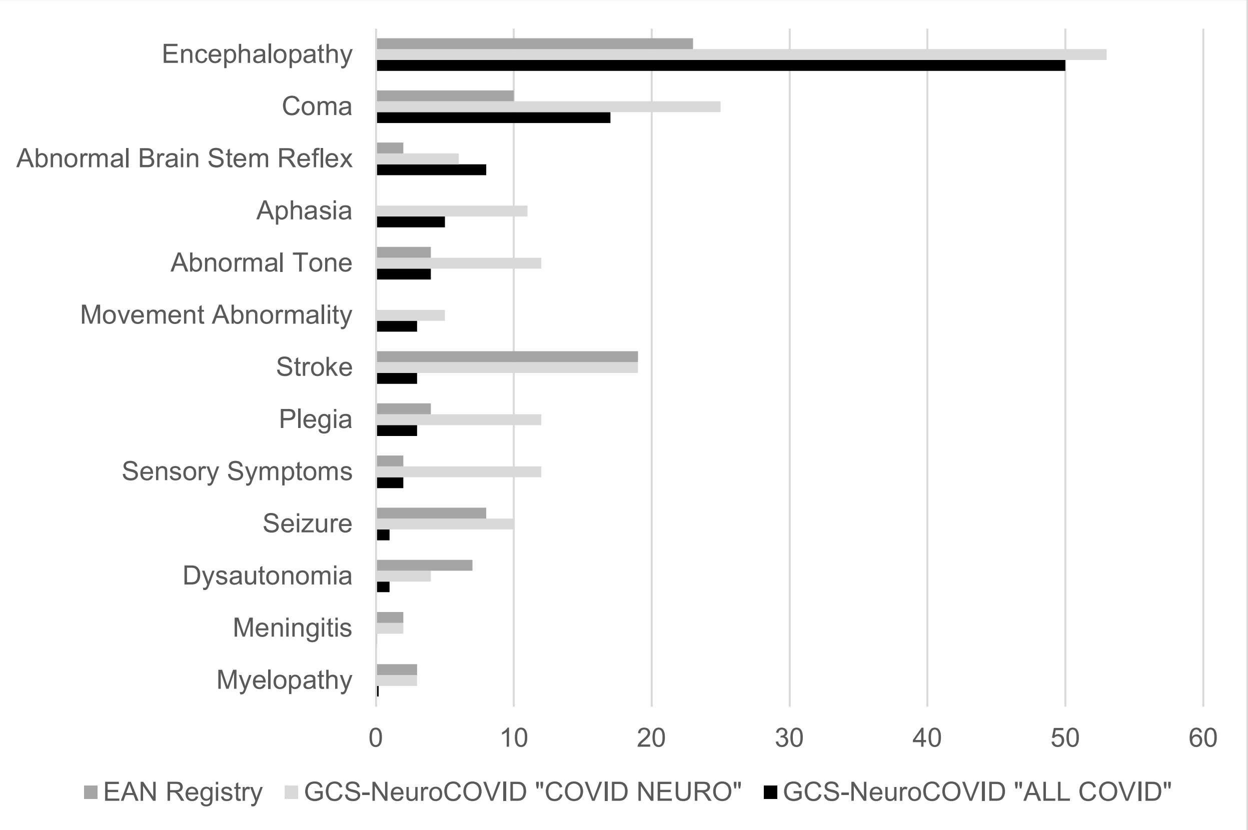

© 2021 Chou SHY et al. *JAMA Network Open.*
