# Supplementary material for: Global Incidence of Neurological Manifestations Among Patients Hospitalized With COVID-19—A Report for the GCS-NeuroCOVID Consortium and the ENERGY Consortium
Source: JAMA Netw Open. 2021 May 11;4(5):e2112131. doi: 10.1001/jamanetworkopen.2021.12131 (PMC8114143; doi:10.1001/jamanetworkopen.2021.12131)
Supplement: Supplement 2. — Nonauthor Collaborators [file jamanetwopen-e2112131-s002.pdf]

Supplemental Online Content: Nonauthor Collaborators

*Indicates required information. Only first name, last name, and suffix will appear in PubMed.

***Group Name(s): GCS-NeuroCOVID Consortium and ENERGY Consortium**

*First Name and Middle Initial(s)	*Last Name	*Suffix (eg, Jr, III)	Academic Degrees	Institution	Location (city, state/province, country)	Role or Contribution, eg, chair, principal investigator	Group (if more than 1 Group listed in the byline) and/or Subgroup (eg, Steering Committee)
Lawrence	Nolan		D.O.	Albany Medical Center	NY, USA	site co-investigator	GCS-NeuroCOVID
Kristi	Temro		M.D.	Albany Medical Center	NY, USA	site co-investigator	GCS-NeuroCOVID
Anna M.	Cervantes-Arslanian		M.D.	Boston Medical Center, Boston University School of Medicine	MA, USA	site principal investigator	GCS-NeuroCOVID
Pria	Anand		M.D.	Boston Medical Center, Boston University School of Medicine	MA, USA	site co-investigator	GCS-NeuroCOVID
Shibani	Mukerji		M.D., PhD	Massachusetts General Hospital Brigham, Harvard Medical School	MA, USA	site principal investigator	GCS-NeuroCOVID
Haitham	Alabasi		M.D.	Massachusetts General Hospital Brigham, Harvard Medical School	MA, USA	site principal investigator	GCS-NeuroCOVID
M. Brandon	Westover		M.D., PhD	Massachusetts General Hospital Brigham, Harvard Medical School	MA, USA	site co-investigator	GCS-NeuroCOVID
Tapan	Kavi		M.D.	Ohio Health Riverside	OH, USA	site principal investigator	GCS-NeuroCOVID
Sayona	John		M.D.	Rush Unversity	IL, USA	site principal investigator	GCS-NeuroCOVID
Ivan	Da Silva		M.D., PhD	Rush Unversity	IL, USA	site co-investigator	GCS-NeuroCOVID
Arif	Shaik		M.D.	United Hospital Allina Health	MN, USA	site principal investigator	GCS-NeuroCOVID
Aarti	Sarwal		M.D.	Wake Forest University	NC, USA	site principal investigator	GCS-NeuroCOVID
Saef	Izzy		M.D., MBChB	Brigham and Women's Hospital, Harvard Medical School	MA, USA	site principal investigator	GCS-NeuroCOVID
Eric M.	Liotta		MD, MS	Northwestern University Feinberg School of Medicine	IL, USA	site principal investigator	GCS-NeuroCOVID
Ayush	Batra		MD	Northwestern University Feinberg School of Medicine	IL, USA	site principal investigator	GCS-NeuroCOVID
Aimee	Aysenne		MD, MPH	Tulane University	LA, USA	site principal investigator	GCS-NeuroCOVID

Supplemental Online Content: Nonauthor Collaborators

*Indicates required information. Only first name, last name, and suffix will appear in PubMed.

*First Name and Middle Initial(s)	*Last Name	*Suffix (eg, Jr, III)	Academic Degrees	Institution	Location (city, state/province, country)	Role or Contribution, eg, chair, principal investigator	Group (if more than 1 Group listed in the byline) and/or Subgroup (eg, Steering Committee)
Clio	Rubinos		MD	University of North Carolina	NC, USA	site principal investigator	GCS-NeuroCOVID
Ahmed Y.	Azzam		MD	Damietta Specialized Hospital, University Faculty of Medicine	Giza, Egypt	site principal investigator	GCS-NeuroCOVID, ENERGY
Mohammed A.	Azab		MD	Cairo University Faculty of Medicine	Cairo, Egypt	site principal investigator	GCS-NeuroCOVID, ENERGY
Justin	Sandall		DO	Ascension Via Christi Hospitals Wichita	KS, USA	site principal investigator	GCS-NeuroCOVID
LeighAnn M.	Persondek		MSN, ACNP-BC, CNRN, ANVP-BC	Ascension Via Christi Hospitals Wichita	KS, USA	site co-investigator	GCS-NeuroCOVID
Hanno	Ulmer		MD	Department of Medical Statistics, Inf	Innsbruck, Austria	Site Investigator	ENERGY
Verena	Rass		MD	Department of Neurology, Neurocritical Care Unit, Medical University of Innsbruck	Innsbruck, Austria	Site Investigator	ENERGY
Bettina	Pfausler		MD	Department of Neurology, Neurocritical Care Unit, Medical University of Innsbruck	Innsbruck, Austria	Site Investigator	ENERGY
Christoph	Müller		MD	Department of Neurology, Neurocritical Care Unit, Medical University of Innsbruck	Innsbruck, Austria	Site Investigator	ENERGY
Simon	Jung		MD	Department of Neurology, University of Bern, Inselspital, Bern, Switzerland	Bern, Switzerland	Site Investigator	ENERGY
Michael	Crean		MS	European Academy of Neurology Headoffice	Vienna, Austria	Manager	ENERGY
Sara	Meoni		MD	CHU Grenoble Alpes	Grenoble, France	Site Investigator	ENERGY
Daniel	Bereczki		MD	Department of Neurology, Semmelweis University	Budapest, Hungary	Site Investigator	ENERGY

*Indicates required information. Only first name, last name, and suffix will appear in PubMed.

***First Name and Middle Initial(s)**	***Last Name**	***Suffix (eg, Jr, III)**	Academic Degrees	Institution	Location (city, state/province, country)	Role or Contribution, eg, chair, principal investigator	Group (if more than 1 Group listed in the byline) and/or Subgroup (eg, Steering Committee)
Tibor	Kovács		MD	Department of Neurology, Semmelweis University	Budapest, Hungary	Site Investigator	ENERGY
Netta	Agajany		MD	Shamir Medical Center	Tel aviv, Israel	Site Investigator	ENERGY
Carmel	Armon		MD	Shamir Medical Center	Tel aviv, Israel	Site Investigator	ENERGY
Sharon	Wolfson		MD	Shamir Medical Center	Tel aviv, Israel	Site Investigator	ENERGY
Maria Sofia	Cotelli		MD	ASST Valcamonica	Brescia, Italy	Site Investigator	ENERGY
Elisa	Bianchi		MD	Istituto di ricerche Farmacologiche Mario negri -_IRCCSS	Milan, Italy	Site Investigator	ENERGY
Anis	Riahi		MD	Military Hospital of Tunis	Tunis, Tunisia	Site Investigator	ENERGY
Şerefnur	Öztürk		MD	Selcuk University	Konya, Turkey	Site Investigator	ENERGY
Onur	Ural		MD	Selcuk University	Konya, Turkey	Site Investigator	ENERGY
Gryb	Viktoriia		MD	Ivano-Frankivsk National Medical University	Ivano-Frankivsk, Ukraine	Site Investigator	ENERGY
Mariana	Lesiv		MD	Ivano-Frankivsk National Medical University	Ivano-Frankivsk, Ukraine	Site Investigator	ENERGY
Luis	Maia		MD	Instituto de Inovação e Investigação em Saúde (I3S), Universidade do Porto , Porto , Portugal & Instituto de Ciências Biomédicas Abel Salazar, Universidade do Porto (ICBAS, UP), Portugal ,Centro Hospitalar Universitário do Porto (CHUP)	Porto, Portugal	Site Investigator	ENERGY
Vanessa	Oliveira		MD	Centro Hospitalar Universitário do Porto (CHUP)	Porto, Portugal	Site Investigator	ENERGY
Mafalda	Seabra		MD	Centro Hospitalar São João (CHUSJ)	Porto, Portugal	Site Investigator	ENERGY
Vanessa	Carvalho		MD	Hospital Pedro Hispano (HPH)	Matosinhos, Portugal	Site Investigator	ENERGY
Paul	Vespa		MD	UCLA, Neurocritical Care Research Central (NCRC)	Los Angeles, USA	Steering Committee Member	GCS-NeuroCOVID

Supplemental Online Content: Nonauthor Collaborators

*Indicates required information. Only first name, last name, and suffix will appear in PubMed.

***First Name and Middle Initial(s)**	***Last Name**	***Suffix (eg, Jr, III)**	Academic Degrees	Institution	Location (city, state/province, country)	Role or Contribution, eg, chair, principal investigator	Group (if more than 1 Group listed in the byline) and/or Subgroup (eg, Steering Committee)
Javier	Provencio		MD PhD	University of Virginia, Neurocritical Care Research Central (NCRC)	Charlottesville, USA	Steering Committee Member	GCS-NeuroCOVID
Daiwai	Olson		RN PhD	University of Texas Southwestern, Neurocritical Care Research Network (NCRN)	Dallas, USA	Steering Committee Member	GCS-NeuroCOVID
Claude	Hemphill		MD	UCSF, Neurocritical Care Research Network (NCRN)	San Francisco, USA	Steering Committee Member	GCS-NeuroCOVID
Chethan P	Venkatasubba Rao		MD	Baylor College of Medicine	Houston, USA	Steering Committee Member	GCS-NeuroCOVID
Nerissa	Ko		MD MS	UCSF	San Francisco, USA	Steering Committee Member	GCS-NeuroCOVID
Ericka	Fink		MD. MS	University of Pittsburgh	Pittsburgh, USA	Pediatrics principal investigator	GCS-NeuroCOVID
Courtney	Robertson		MD	Johns Hopkins University	Baltimore, USA	Pediatrics principal investigator	GCS-NeuroCOVID
Michelle	Schober		MD	University of Utah	Salt Lake City, USA	Pediatrics principal investigator	GCS-NeuroCOVID
Ali	Smith Scott		BS	University of Pittsburgh	Pittsburgh, USA	Consortium project manager	GCS-NeuroCOVID
Michal	Hammond		MD	Ascension Genesys Hospital	Michigan, USA	Consortium research coordinator	GCS-NeuroCOVID
Nicole	Paul		BS	University of Pittsburgh	Pittsburgh, USA	student co-investigator	GCS-NeuroCOVID
Aleksandra	Safonova		BS	University of Pittsburgh	Pittsburgh, USA	student co-investigator	GCS-NeuroCOVID
Lauren	Kaplan		BS	University of Pittsburgh	Pittsburgh, USA	student co-investigator	GCS-NeuroCOVID
Charith	Ratnayake		BS	University of Pittsburgh	Pittsburgh, USA	student co-investigator	GCS-NeuroCOVID

Supplemental Online Content: Nonauthor Collaborators

*Indicates required information. Only first name, last name, and suffix will appear in PubMed.

***First Name and Middle Initial(s)**	***Last Name**	***Suffix (eg, Jr, III)**	Academic Degrees	Institution	Location (city, state/province, country)	Role or Contribution, eg, chair, principal investigator	Group (if more than 1 Group listed in the byline) and/or Subgroup (eg, Steering Committee)
Adytia D	Sharma			University of Pittsburgh	Pittsburgh, USA	student co-investigator	GCS-NeuroCOVID
Abigail	Skeel			University of Pittsburgh	Pittsburgh, USA	student co-investigator	GCS-NeuroCOVID
Carlos	Villamizar Rosales		MD MS	University of Pittsburgh	Pittsburgh, USA	site co-investigator	GCS-NeuroCOVID
Dominika	Dolak		MD	University of Pittsburgh	Pittsburgh, USA	site co-investigator	GCS-NeuroCOVID
Panayiotis	Varelas		MD PhD	Albany Medical Center	NY, USA	site principal investigator	GCS-NeuroCOVID
Lev	Lotman		MD	Albany Medical Center	NY, USA	site co-investigator	GCS-NeuroCOVID
Lalit	Kaltenbach		BS	Medical University Innsbruck	Innsbruck, Austria	consortium informatics	ENERGY
Menon	David K.		MD PhD	University of Cambridge	Cambridge, UK	co-investigator	GCS-NeuroCOVID
